# Reduced apple crop density enhances total polyphenol accumulation via upregulation of anthocyanidin reductase and other phenylpropanoid pathway genes

**DOI:** 10.3389/fpls.2025.1591292

**Published:** 2025-06-09

**Authors:** Shanthanu Krishna Kumar, Kamal Tyagi, Michael Brown, Lailiang Cheng, Zhangjun Fei, Gregory Peck

**Affiliations:** ^1^ Department of Plant Science, College of Agricultural Sciences, The Pennsylvania State University, University Park, PA, United States; ^2^ School of Integrative Plant Science—Horticulture Section, College of Agriculture and Life Sciences, Cornell University, Ithaca, NY, United States; ^3^ Boyce Thompson Institute, Ithaca, NY, United States

**Keywords:** crop load, hard cider, *Malus* ×*domestica*, phenolics, tannin

## Abstract

Polyphenols contribute to the quality of hard cider fermented from apple (*Malus* ×*domestica*) juice by providing flavor, aroma, color, and microbial stability. However, polyphenol concentration in apple fruit can fluctuate by 50% or more from tree-to-tree within an orchard of the same scion and rootstock resulting in significant year-to-year product variability. In order to better understand polyphenol biosynthesis in cider apples, four-year-old ‘Porter’s Perfection’ and ‘Binet Rouge’ trees were left unthinned (control), or had fruitlets adjusted to low, medium, or high crop density. Fruit peel and flesh tissue were sampled at 27, 81, and 160 (harvest) days after full bloom (DAFB) and analyzed for polyphenol concentration and composition, as well as gene expression. At 160 DAFB, there was a 39% increase in monomeric and oligomeric polyphenol concentrations in the ‘Porter’s Perfection’ flesh tissue of the reduced crop density treatments as compared to the unthinned control. The transcriptome profile of the low crop density ‘Porter’s Perfection’ treatment indicated that genes encoding enzymes that catalyze critical functions in the phenylpropanoid pathway such as hydroxylation, methylation, and glycosylation were upregulated compared to the control at 27 DAFB and 81 DAFB. The period of upregulated gene expression corresponded with increased concentration of polyphenols, particularly proanthocyanidin monomers and oligomers. Specifically, there was a significant increase in anthocyanidin reductase (an enzyme involved in epicatechin catalysis) expression in the low crop density treatment relative to the unthinned control at 27 and 81 DAFB in both the peel and flesh. Reduced crop densities enhanced the expression of genes involved in the phenylpropanoid pathway in apples, which likely increased fruit polyphenols. Furthermore, we identified eight and three novel ethylene response factor genes, 26 and 14 MYB-bHLH genes in the flesh and peel, respectively, that are potentially involved in regulating proanthocyanidin biosynthesis. These data suggest that reduced crop load densities lead to enhanced polyphenol synthesis and accumulation in ‘Porter’s Perfection’ apples via transcriptional regulation of anthocyanidin reductase and other genes in the phenylpropanoid pathway.

## Introduction

Hard cider is an alcoholic beverage made by fermenting apple (*Malus* ×*domestica*) juice. Cider apple cultivars have fruit characteristics that focus on juice chemical concentration and composition, particularly of polyphenols but also organic acids and sugars, more so than the cosmetic appearance, size, and texture that is important for fresh-market apples. Understanding polyphenol biosynthesis in apples and the management practices that affect the production of these compounds will aid apple growers and cider producers in increasing product quality.

Polyphenols are grouped into five categories: dihydrochalcones (e.g., phloridzin), phenolic acids (e.g., chlorogenic acid and hydroxycinnamic acids), flavonols (e.g., quercetin glycosides), proanthocyanidins (e.g., catechin, epicatechin, and their oligomers and polymers), and anthocyanins (e.g., cyanidin glycosides) (Takos et al., 2006a; Ban et al., 2007). Flavonols and anthocyanins are mostly present in peel tissue and are not extractable during typical fruit processing and fermentation practices (Guyot et al., 2003; Devic et al., 2010), although there are a few red fleshed genotypes used in rosé cider production (Renard et al., 2007). Little is known about the impact of dihydrochalcones on cider flavor, aroma, or fermentation process. Phenolic acids such as hydroxycinnamic acids can slow fermentation rates and influence perceptible aroma characteristics (Cairns et al., 2021). Hydroxycinnamic acids also disintegrate into ethylphenol compounds which produce what Chatonnet et al. (1992) referred to as “barnyard” or “leather” aromas, which are characteristic in cider apple cultivars such as ‘Kingston Black’ as well as other cidermaking styles (Whiting, 1975). Proanthocyanidins, also known as condensed tannins, are mostly in fruit flesh and are one of the most important polyphenol categories because they are directly related to organoleptic quality. When proanthocyanidin oligomers and polymers are under four sub-units long they tend to taste bitter, whereas larger tannin units are responsible for an astringent mouthfeel in cider (Lea and Arnold, 1978). Fresh-market apples possess low concentrations of proanthocyanidins (<100 mg·L^-1^), whereas many cider apples have greater than 2 g·L^-1^ (Renard et al., 2007).

Through the phenylpropanoid pathway, the monomers catechin and epicatechin are produced due to the activity of leucoanthocyanidin reductase (*LAR*) and anthocyanidin reducatase (*ANR*), respectively, which can then polymerize with catechin initiators and epicatechin elongators to form proanthocyanidin oligomers (Lea and Arnold, 1978; Guyot et al., 2003; Henry-Kirk et al., 2012). Many recent studies have focused on transcription factors (TFs) and their role in regulating the phenylpropanoid pathway. The myeloblastosis (MYB) family of genes plays a major role in regulation of the phenylpropanoid pathway through transcriptional activation or repression of the expression of key structural genes in the phenylpropanoid pathway (Li et al., 2015). They work in conjunction with other TFs such as the basic helix loop helix protein (bHLH) and beta-transducin proteins (WD) (Allan et al., 2008). Ethylene response factors (ERFs) also play a critical role in the phenylpropanoid pathway. In particular, *MdERF1B* and *MdERF3* have been identified to promote proanthocyanidin and anthocyanin synthesis together with MYB genes such as *MdMYB1* and *MdMYB11* (Zhang et al., 2018). Additionally, *MdRAV1* was found to bind to the promoter of *MdANR2* (anthocyanidin reductase) and inhibit its activity (Li et al., 2020). Other studies have indicated that *MdERF1A*, *1B*, and *MdERF23* contribute to procyanidin regulation (Zhang et al., 2018; Li et al., 2020). The transcriptional and epigenetic control of these phenylpropanoid genes in response to physiological factors such as crop density has not yet been investigated.

The effect of crop density on low polyphenol fresh-market apple cultivars has mostly focused on peel anthocyanins or flesh antioxidants (Awad et al., 2001; Wünsche et al., 2005; Peck et al., 2016). Zakalik et al. (2023) conducted a three-year study on the effect of crop density on seven cider apple cultivars juice quality parameters such as soluble solids, acidity, and total polyphenol concentration. Results from that study found that while polyphenol concentrations increased with crop density in the first year of treatment implementation, cumulative yields of polyphenols over a three-year period decreased with increased crop density, while reducing biennial bearing tendencies (Zakalik et al., 2024). That study recommended a crop density of ~9 fruits/cm^2^ trunk cross sectional area (TCSA) to maximize yields and polyphenol concentrations while maintaining required return bloom for the next year, which are greater than fresh-market crop density recommendations (Robinson et al., 2023).

The aim of this experiment was to understand the effect of crop density on the physiological and molecular underpinnings of polyphenol biosynthesis in cider apples. Phenylpropanoid pathway metabolites were also studied throughout the growing season to characterize the polyphenol accumulation patterns. We hypothesize that most polyphenolics would be produced within 35 DAFB and subsequently be reduced in concentration with a corresponding increase in fruit size. We also hypothesized that reduced crop density would enhance polyphenol production in cider apples, especially catechin and epicatechin biosynthesis with a corresponding upregulation in key phenylpropanoid genes under reduced crop density.

## Materials and methods

### Trial location and experimental design

The experiment was conducted in 2021 at the Cornell University Agricultural Experiment Station, Ithaca, NY (42.443880, -76.464919) using two commercially important high-tannin cider cultivars (Peck et al., 2021). According to the Long Ashton Research Station classification system, ‘Porter’s Perfection’ is an English bittersharp (high in both tannins and acidity) and ‘Binet Rouge’ is a French bittersweet (high in tannins and low in acidity) (Barker and Ettle, 1910). All trees used in this experiment were grafted onto ‘Geneva 11’ (‘G.11’) rootstock and planted in spring 2018 at 1.2 m between trees and 3.7 m between rows (~2,200 trees/ha) in uniform rows of approximately 100 trees. The trees were trained as tall spindles having a single dominant central leader attached to a 4-wire trellis. The orchard was managed using a conventional pest management regimen for diseases and arthropods (Agnello et al., 2021).

Five replicated blocks each contained four crop density treatments: low (5 fruit/cm^2^ TCSA), medium (10 fruit/cm^2^ TCSA), high (15 fruit/cm^2^ TCSA), and an unthinned control treatment (UTC), which had no crop density manipulation. Two trunk diameter measurements were taken perpendicular to each other at 40 cm above the graft union to measure TCSA. Each of the four treatments were randomly assigned to two side-by-side trees within each block. The TCSA for each tree was multiplied by the target crop density to determine the fruit number per tree. Fruit clusters were first thinned to the central fruit in the cluster and then the trees were hand thinned to arrive at the calculated fruit number. Efforts were made to uniformly distribute the fruit throughout the canopy. Trees were assessed to be in full bloom (greater than 50% of the flowers in bloom) on 14 May 2021 for ‘Porter’s Perfection’ and on 20 May 2021 for ‘Binet Rouge’. The hand thinning treatments were implemented on 27 May 2021 for ‘Porter’s Perfection’ and on 1 June 2021 for ‘Binet Rouge’. No chemical flower or fruit thinning chemicals were applied.

### Fruitlet and harvest sampling, and return bloom assessment

Six representative fruit from each experimental unit were sampled throughout the growing season at approximately 4-week intervals of 20, 48, 74, 105, and 146 days after full bloom (DAFB) for ‘Binet Rouge’, and at 27, 55, 80, 110, 139, and 160 DAFB for ‘Porter’s Perfection’. The fruit samples were collected and kept on ice until they were separated into peel and cortex tissue using knives. Care was taken to remove the seed region including all the seeds from the tissue samples. Fruit tissue samples were flash frozen in liquid nitrogen and then stored at -80°C until analysis.

Using an iodine solution consisting of 0.22 g·L^−1^ iodine, 0.88 g·L^−1^ potassium iodide (Sigma-Aldrich, St. Louis, MO, USA) the Starch Pattern Index (SPI) was used to assess the maturity of fruit to decide the harvesting date, and fruit were harvested as close to an SPI of 6 out of 8, meaning most of the starch had been hydrolyzed to sugars (Blanpied and Silsby, 1992). ‘Porter’s Perfection’ was harvested on 23 Oct 2021 at an average SPI of 6.9. However, ‘Binet Rouge’ experienced a heavy pre-harvest fruit drop and had to be harvested earlier than full maturity on 7 Oct 2021 with an average SPI of 3.1. Fruit yields were recorded for each experimental tree. Fruit mass was measured with an Adam CPW field scale (Oxford, CT, USA). Return bloom was assessed on 17 May 2022 for ‘Porter’s Perfection’ and on 19 May 2022 for ‘Binet Rouge’ by counting the number of flower clusters on each experimental tree at the “pink” stage of flower development (Chapman and Catlin, 1976).

### Fruit quality analyses

A subset of ten fruit per experimental unit were randomly selected and used for maturity and quality analyses. Fruit mass and diameter were measured using a caliper integrated into a GÜSS Fruit Texture Analyzer (Jennings, Strand, South Africa). Visual color measurements of fruit were taken, expressed as percent surface area of the fruit covered by red blush, with the parameter ranging from 0-100%. Peel chlorophyll content was measured as a proxy for ripeness using the degree of absorbance meter (Model 53500, T.R. Turoni Srl, Forli, Italy). Fruits were then assessed for flesh firmness on both the blush and non-blush side with a penetrometer (GÜSS Fruit Texture Analyzer) fitted with an 11.1mm probe. Subsequently, fruit ripeness was measured using the SPI by spraying an equatorial wedge of the fruit with the iodine solution. Following the firmness and ripeness testing, fruit were milled, placed into Good Nature filter bags (Buffalo, NY, USA), and pressed using the Norwalk 290 tabletop juicer (Bentonville, AR, USA). This set up is similar to a rack and cloth style press used by many cider producers. Juice samples were then frozen at -80°C until further analyses.

### Juice chemistry analysis

Samples were analyzed for soluble solids content (SSC), titratable acidity (TA), and total polyphenol content (TPC). SSC was measured using a hand-held PAL-1 BLT digital refractometer (Omaeda, Saitama, Japan). TA was measured using a Metrohm 809 Titrando autotitrator (Herisau, Switzerland) by titrating 5 mL of juice in 40 mL of ultrapure Milli-Q-water (Darmstadt, Germany) against a 0.1 M NaOH solution (Sigma-Aldrich, St. Louis, MO, USA) until the pH reached a value of 8.1. TPC was measured using the Folin-Ciocalteau method (Singleton and Rossi, 1965) on a Spectramax 384 Plus spectrophotometer and Softmax Pro 7 Microplate Data analysis software (Molecular Devices, San Jose, CA). For juice samples, 1.5 µL of the sample or gallic acid standard (Sigma-Aldrich, St. Louis, MO, USA) was mixed with 34.9 µL of water and 90.9 µL of Folin-Ciocalteau reagent (M.P. Biochemicals, Aurora, Ohio, USA) in a Cellistar 96 well microplate (Greiner Bio-One, Monroe, NC, USA); six min after, 72.7 µL of 7% (w/v) Na_2_CO_3_ (Sigma-Aldrich, St. Louis, MO, USA) was added; the microplate was incubated for 1 hr. under dark conditions before being measured at 765 nm. Gallic acid was used as a standard for the TPC measurement and results were reported in gallic acid equivalents.

For peel and flesh tissue, TPC was measured according to the protocol developed by Huber and Rupasinghe (2009) with a few modifications. The flash frozen apple peel and cortex tissue stored at -80°C were lyophilized in a freeze drier (Labonco FreeZone 12L-84C bulk tray drier, Kansas City, MO, USA) which was set to 0°C for 72 hr., and ground to a fine powder using a coffee grinder (KitchenAid, Benton Harbor, MI, USA). Two hundred milligrams of the tissue sample were sonicated (Branson 2800, Sonitek, Milford, CT, USA) with 10 mL of HPLC grade 100% methanol (VWR Chemicals, Radnor, PA, USA) for 15 min to extract the polyphenols. The extract was then centrifuged at 3,500 ×g for 15 min. Ten microliters of the extract, or gallic acid was mixed with 100 µL of the Folin-Ciocalteau reagent and gently mixed in a 96-well clear microplate. After 6 min, 80 µL of 7.5% (w/v) Na_2_CO_3_ was added followed by a 1-hr incubation period in the dark and absorption measured at 765 nm.

### High performance liquid chromatography analysis

Polyphenol analysis was performed using a Reverse Phase-HPLC on harvested juice samples, as well as lyophilized peel and cortex samples collected throughout the growing season, using a protocol modified from Hendrickson et al. (2016) and Tyagi et al. (2020). The juice samples were diluted in a 1:1 ratio with 100% methanol (containing 0.1% ascorbic acid and 0.1% hydrochloric acid), vortexed, and centrifuged at 20,000 g for 10 min at 4°C. The supernatant was again centrifuged at 20,000 ×g for 15 min at 4°C before injection.

Lyophilized powder (100 mg) of peel and cortex was homogenized in 3 mL 50% (v/v) methanol solution (containing 0.1% v/v hydrochloric acid and 0.1% w/v ascorbic acid) to extract monomeric and oligomeric polyphenols. Homogenates were vortexed and incubated at 4°C for 4 hr. on ice with occasional mixing and were centrifuged at 4,000 g for 5 min. Pellets were reextracted twice with 1 mL each time and all the supernatants were combined brought to the final volume of 5 mL. From this supernatant, 1 mL was centrifuged at 14,000 g for 5 min at 4°C to remove any particulates and 5 μL was injected into the HPLC column for analysis. The remaining 4 mL of sample was stored at -20°C for proanthocyanidin (PA) measurements.

Polyphenol monomers and oligomers were chromatographically separated by RP-HPLC using a Poroshell HPH-C18 column (4.6 ×100 mm, 2.7 μm particle size) on an Agilent Infinity series 1260 HPLC system (Agilent Technologies, Santa Clara, CA, USA). The system was equipped with a diode array detector (DAD) and operated using a binary solvent gradient with mobile phase A [1.5% (v/v) formic acid in ultrapure Milli-Q water] and mobile phase B [1.5% (v/v) formic acid and 1.4% (v/v) water in acetonitrile] with a flow rate on 1 mL min-1. A 10 µL sample was injected into the column for analysis and the column temperature was maintained at 35°C. The starting condition of the gradient was 95% of solvent A and 5% of solvent B. Subsequently, solvent B was linearly increased to 15% in 25 min, then to 27% in 10 min, and kept at 27% for 3 min. Thereafter, the mobile phase was reverted to the initial condition in 2 min and held for 3 min for re-equilibration of the column before the next injection, for a total run time of 43 min.

Avicularin, 4-caffeoylquinic acid, 5-caffeoylquinic acid, (+)-catechin, chlorogenic acid (3-caffeoylquinic acid), p-coumaric acid, (−)-epicatechin, ferulic acid, phlorizin, procyanidin A1, procyanidin A2, procyanidin B2, and procyanidin C1, quercetin derivatives [hyperoside (quercetin-3-galactoside), isoquercetin (quercetin-3-glucoside), quercitrin, rutin (quercetin-3-O-rutinoside) and sinapic acid were purchased from Sigma-Aldrich. Procyanidin B1 was purchased from INDOFINE Chemical Company (Hillsborough, NJ, USA). The DAD wavelengths were set at 280 nm [(+)-catechin, (−)-epicatechin, procyanidin A1, procyanidin A2, procyanidin B1, procyanidin B1, procyanidin C1, and phlorizin], 320 nm (4-caffeoylquinic acid, 5-caffeoylquinic acid, chlorogenic acid, p-coumaric acid, ferulic acid, and sinapic acid), and 360 nm (avicularia, hyperoside, isoquercetin, rutin, and quercitrin). The eluted compounds were monitored and identified by comparing spectral and retention time to external standards. The identified compounds were quantified by external calibration curves. All data processing and analysis were completed using Agilent CDS ChemStation software on an Agilent 1260 Infinity RP-HPLC.

### Proanthocyanidin extraction and phloroglucinol analysis

The proanthocyanidin (PA) analysis of lyophilized peel and cortex tissue was processed and analyzed according to the protocol developed by Tyagi et al. (2020). The pellet from the polyphenol monomers and oligomers extraction were resuspended in 8 mL of 70% acidified acetone (containing 0.1% ascorbic acid and 0.05% trifluoroacetic acid) for the extraction of polymers. The acetone extract was centrifuged at 4,000 g for 5 min after overnight extraction at 4°C with occasional mixing. The pellet was re-extracted twice in 2 mL of acidified acetone to make a final volume of 12 mL. The methanol supernatant from the polyphenol analysis was combined with the acetone supernatants and was evaporated under vacuum at 34°C to near dryness using a Buchi^®^ Rotavapor R110 (Flawil, Switzerland). The extract was resuspended in 1 mL of methanol and stored at −20°C until further analysis.

Proanthocyanidins were isolated by solid phase extraction (SPE) as describe by Girardello et al. (2019). Bed columns containing 10 mL of Toyopearl HW-40 F size exclusion media (Tosoh Biosciences, Shiba, Tokyo, Japan) were used to purify the proanthcyanidins and SPE was performed in triplicate with two technical replications. Before loading to the SPE column, all methanol extract were diluted with water to reach a concentration of 25% methanol. The eluates from the SPE columns containing isolated PAs were concentrated under reduced pressure and dissolved in 500 μL methanol and stored at −80°C until analysis.

The composition of isolated PAs from SPE extraction was determined by phloroglucinolysis (Kennedy and Jones, 2001) with some modifications (Tyagi et al., 2020). Briefly, 80 μL of sample and 80 μL of phloroglucinol reagent (100 mg·mL-1 phloroglucinol (Sigma-Aldrich, St. Louis, MO, USA) and 20 mg·mL-1 ascorbic acid (Avantor, Radnor, PA, USA) prepared in 0.2 M HCl acidified methanol (Sigma-Aldrich, St. Louis, MO, USA) were combined and vortexed. The reaction was performed in duplicate at 50°C for 20 min and it was quenched with 800 μL of 40 mM sodium acetate (VWR Chemicals, Solon, Ohio, USA). Quenched samples were centrifuged at 14,000 ×g for 5 min to sediment any debris or particles. The phloroglucinolysis reaction products were chromatographically separated by RP-HPLC using a Kinetex C18 (4.6 × 150 mm, 2.6 μm particle) HPLC column on the HPLC machine described above using a binary solvent gradient with mobile phase A (0.1% formic acid in water) and mobile phase B (0.1% formic acid in acetonitrile). Column temperature was maintained at 35°C. The diode array detector wavelength was set to 280 nm. Injection volume of 20 μL was used for extracted sample. The starting condition of the gradient was 94% of solvent A and 6% of solvent B. Subsequently, solvent B was linearly increased to 18% in 13 min, then to 85% in 1 min and kept at 85% for 2 min. Thereafter, the mobile phase was reverted to the initial condition in 1 min and held for 3 min for re-equilibration of the column before the next injection. Data analysis was performed using Agilent CDS ChemStation software and individual reaction products (Epicatechin-phloroglucinol adduct, catechin, and epicatechin) were quantified by an external calibration curve for (+)-catechin (C) using their response factor relative to catechin (Singleton and Rossi, 1965).

### RNA extraction and library preparation

The frozen peel and cortex tissue was finely ground under liquid nitrogen using a pestle and mortar. RNA was extracted from the peel and cortex tissue using the protocol developed by Reid et al. (2006). The finely ground tissue samples (350 mg) were added to 10 mL of pre-warmed (65°C) RNA extraction buffer (2% CTAB, 2% PVP, 300 mM Tris HCl at pH 8.0, 25 mM EDTA, 2 M NaCl, 0.05% spermidine trihydrochloride, 2% β-mercaptoethanol), and incubated in 65°C water bath for 10 min with regular vortexing. All the above chemicals were obtained from VWR Chemicals (Solon, Ohio, USA). Ten milliliters of chloroform (VWR Chemicals, Radnor, PA, USA): isoamylalcohol (VWR Chemicals, Solon, Ohio, USA) in the ratio of 24:1 was added to the mixture and centrifuged at 3,500 ×g for 15 min at 4°C. The aqueous layer was transferred to a new tube and centrifuged at 30,000 ×g for 20 min at 4°C to remove any remaining insoluble material. This step was repeated twice, following which 0.1 vol 3 M sodium acetate (pH 5.2) and 0.6 vol isopropanol (Avantor, Radnor, PA, USA) were added, mixed, and then stored at -80°C for 30 min. Nucleic acid pellets (including any remaining carbohydrates) were collected by centrifugation at 3,500 ×g for 30 min at 4°C. The pellet was dissolved in 1 ml Tris-EDTA [pH 7.5, VWR Chemicals, Radnor, PA, USA)] and transferred to a microcentrifuge tube. To selectively precipitate the RNA, 0.3 mL of 8 M LiCl (VWR Chemicals, Solon, Ohio, USA) was added and the sample was stored overnight at 4°C. The RNA was pelleted by centrifugation at 20,000 ×g for 30 min at 4°C then washed with 500 µL of ice cold 70% EtOH (VWR Chemicals, Radnor, PA, USA), air dried, and dissolved in 5100 μl DEPC-treated water. The RNA was checked for purity using the 260/280 nm ratios on a NanoDrop ND-1000 spectrophotometer (NanoDrop Technologies, Wilmington, DE, USA), and check for integrity using electrophoresis with a 1% agarose gels and GelDoc (Bio-Rad, Hercules, California, USA). RNA was purified using a RNeasy kit (Qiagen, Valencia, CA, USA) and rechecked for purity.

Gene expression and transcriptome analysis were conducted by Polar Genomics LLC. (Ithaca, NY, USA). Strand-specific RNAseq libraries were constructed using the protocol described by Zhong et al. (2011). Briefly, the steps included polyA RNA isolation and fragmentation, first-strand complementary DNA (cDNA) synthesis, second strand synthesis with Deoxyuridine Triphosphate (dUTP), end-repair, DNA A-tailing, Y-shape adapter ligation, triple-solid-phase reversible immobilization purification and size selection, PCR enrichment, and mixed barcoded libraries for multiplexed sequencing.

### RNA sequencing, differential gene expression, and gene enrichment analysis

Pooled libraries were sequenced using HiSeqX 150 bp paired end sequencing (Psomagen Inc, Rockville MD). Raw RNA-Seq sequencing reads have been deposited in the NCBI BioProject database under the accession number PRJNA1004866. Raw RNA-Seq reads were processed to remove adaptors and low-quality sequences using Trimmomatic (version 0.39; Bolger et al., 2014) with parameters ‘SLIDINGWINDOW:4:20 LEADING:3 TRAILING:3 MINLEN:40’ and to remove polyA/T tails using PRINSEQ++ (v1.2; Cantu et al., 2019) with parameters ‘-min_len 40 -trim_tail_left 10 -trim_tail_right 10’. The remaining cleaned reads were aligned to the ribosomal RNA database (Quast et al., 2013) using Bowtie (version 1.1.2; Langmead, 2010) allowing up to three mismatches, and those aligned were discarded. The final cleaned reads were aligned to the ‘Golden Delicious’ double haploid (GDDH13) genome (v1.1; Daccord et al., 2017) using HISAT2 (version 2.1.0; Kim et al., 2019) with default parameters. Based on the alignments, raw read counts for each gene were calculated and then normalized to fragments per kilobase of exon model per million mapped fragments (FPKM). Raw read counts were then fed to DESeq2 to identify differentially expressed genes (DEGs) using a cutoff of adjusted *P*-value < 0.05 and fold change ≥ 2. Gene ontology terms enriched in the lists of genes were identified using Blast2GO (Conesa et al., 2005) with a cutoff of adjusted *P*-value < 0.05. TFs and transcriptional regulators were identified by using the iTAK program (Zheng et al., 2016).

### Statistical analysis

This experiment was analyzed as a randomized complete block design using R statistical software (R Core Team, 2014). Regressions were analyzed as mixed models with a random block term, using the lmer function from the lme4 package. The emtrends function was used to separate the regression trendlines according to cultivar. Mean separation for a family of estimates (estimated marginal means, emmeans package), using the Tukey method, was performed using the cld function (multcomp package). Model assumptions were checked by assessing R^2^ values and examining the distribution and spread of residuals. The ggplot2 function was used to plot regressions and estimated marginal means graphs. A heatmap of the individual polyphenol monomers was generated with the Metaboanalyst 5.0 (Pang et al., 2021) software with the autoscaling function (mean-centered and divided by the square root of the standard deviation of each variable). The Venn diagrams were generated with Venny 2.1 (Oliveros, 2007-2015).

## Results

### Yield and return bloom

‘Porter’s Perfection’ had an average TCSA of 14.7 cm^2^ and ‘Binet Rouge’ had an average TCSA of 7.8 cm^2^ (**Supplementary Figure S1**). There were no significant differences in TCSA among treatments for either cultivar. The ‘Porter’s Perfection’ trees were larger in size and thus had a greater yield than the ‘Binet Rouge’ trees. There was a significant positive linear correlation between crop density and yield for both ‘Porter’s Perfection’ (*P* < 0.0001) and ‘Binet Rouge’ (*P* < 0.0001). The UTC treatments had the greatest yields at 32.6 and 8 kg/tree for ‘Porter’s Perfection’ and ‘Binet Rouge’, respectively. Return bloom density had a significantly negative linear correlation with crop density for both ‘Porter’s Perfection’ (*P* < 0.0001) and ‘Binet Rouge’ (*P* = 0.0032). For both cultivars, the UTC treatments had no flower blooms in 2022. Thus, despite producing high yields during the experiment year (2021), there would have been no fruit the next year. Based on the regression model, ‘Porter’s Perfection’ would have zero return bloom the next year when the crop density was equal to or greater than 28.4 fruit/cm^2^ TCSA, whereas ‘Binet Rouge’ would not have any return bloom once there were more than 27.8 fruit/cm^2^ TCSA.

### Fruit mass and diameter

**Figure 1:**
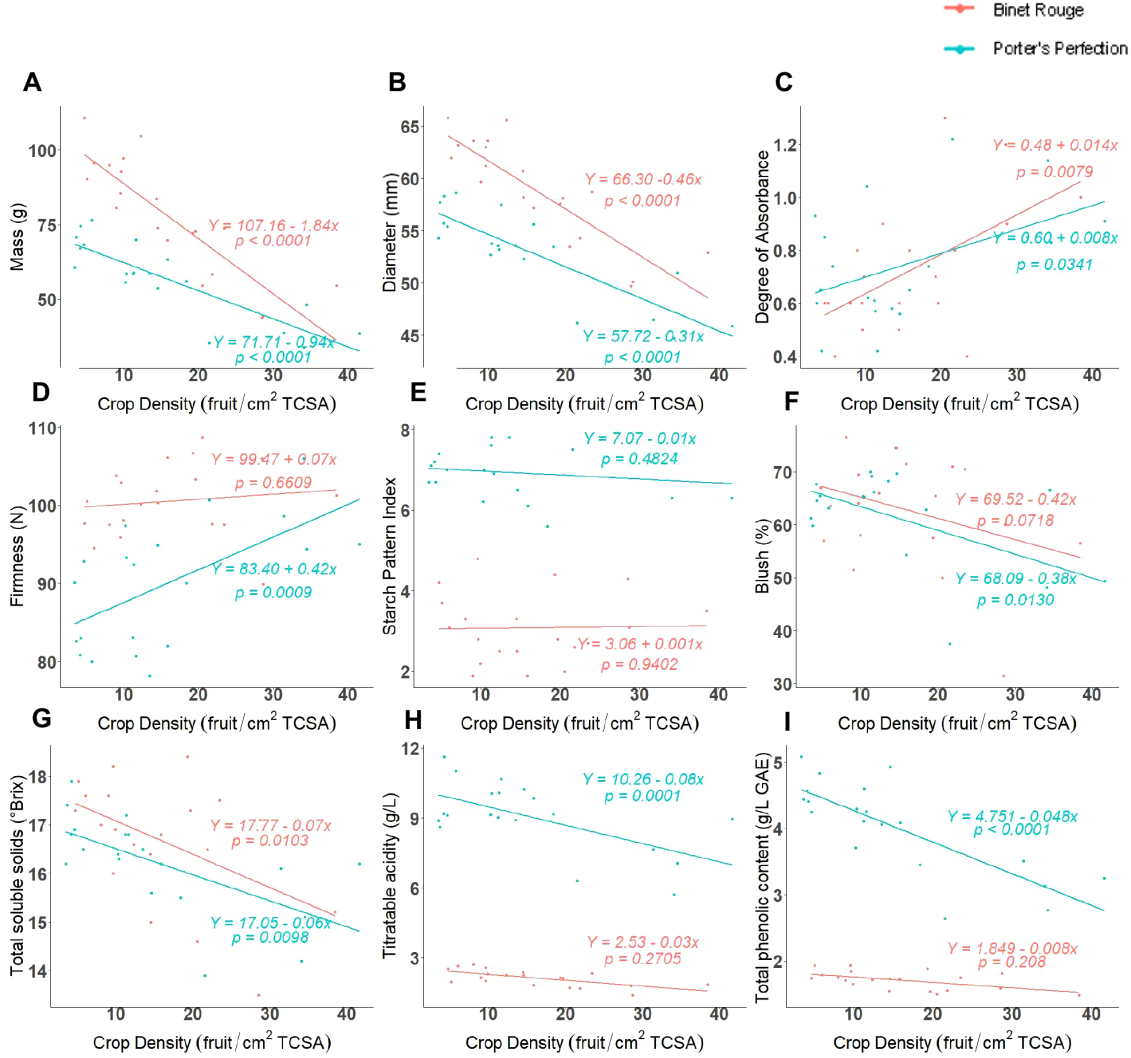
Regression between crop density [fruit per trunk cross-sectional area (TCSA)] and at harvest fruit and juice measurements for ‘Porters Perfection’/’G.11’ and ‘Binet Rouge’/’G.11’ trees subjected to four crop density treatments. Each datapoint represents a mean value **(A-F)** or a composite juice sample **(G-I)** for 10 apples per experimental unit.

The individual fruit mass of both cultivars had a significant negative linear correlation with crop density (**Figure 1A**, P < 0.0001). ‘Binet Rouge’ had a stronger inverse relationship with fruit mass as compared to ‘Porter’s Perfection’. ‘Porter’s Perfection’ and ‘Binet Rouge’ exhibited a 0.9 and 1.8 g decrease, respectively, for every unit (fruit/cm^2^ TCSA) increase in crop density. The individual average fruit mass of ‘Porter’s Perfection’ and ‘Binet Rouge’ across all treatments was 57 and 78 g respectively. Similarly, fruit diameter had a significant negative linear relationship with crop density for both cultivars (**Figure 1B**, P < 0.0001).

### Fruit maturity and quality parameters

The difference in absorbance (DA), blush, starch pattern index (SPI), and flesh firmness were used to assess fruit maturity and quality. The greater the DA value, the more chlorophyll is present and thus the fruit is less mature. The DA values for both cultivars ranged between 0.6 and 1.2 (**Figure 1C**). The DA values for both cultivars were had a positive linear relationship with crop density. This relationship was significant for ‘Porter’s Perfection’ (*P* = 0.0300).

There was a marked difference in the relationship between firmness and crop density for both cultivars. While ‘Porter’s Perfection’ had a significant positive linear relationship with firmness (*P* = 0.0009), ‘Binet Rouge’ had an insignificant increase in firmness with greater crop density (**Figure 1D**). On average, ‘Porter’s Perfection’ had a firmness of 90 N, whereas ‘Binet Rouge’ had a greater firmness at 101 N. The SPI varied significantly between the two cultivars. ‘Porter’s Perfection’ was harvested at an average SPI of 6.9, whereas ‘Binet Rouge’ was harvested at 3.1 SPI due to pre-harvest fruit drop (**Figure 1E**). Some cultivars such as ‘Binet Rouge’ are prone to pre-harvest fruit drop before fruits are fully mature, and we had to harvest the fruit left on the tree earlier than recommended to obtain fruits for our analyses. Both cultivars had an insignificant relationship between SPI and crop density. Both ‘Porter’s Perfection’ and ‘Binet Rouge’ had ~60% red peel blush pigmentation (**Figure 1F**). ‘Porter’s Perfection’ had a significant negative correlation between blush and crop density (*P* = 0.0130).

### Juice quality parameters

Total soluble solids (TSS), titratable acidity (TA), and total polyphenol concentration (TPC) were measured on juice extracted from different crop density treatments at harvest. Both cultivars had an average TSS content of ~16°Brix (**Figure 1G**). Both ‘Porter’s Perfection’ and ‘Binet Rouge’ had a significant negative linear relationship of TSS with crop density (*P* = 0.0098 for ‘Porter’s Perfection’ and *P* = 0.0103 for ‘Binet Rouge’). ‘Porter’s Perfection’ had an average TA of 9 g·L^-1^, whereas ‘Binet Rouge’ had an average TA of 2.1 g·L^-1^ (**Figure 1H**). ‘Porter’s Perfection’ had a significant negative linear relationship between TA and crop density (*P* < 0.0001). ‘Porter’s Perfection’ had an average TPC of 4 g·L^-1^, whereas ‘Binet Rouge’ had an average TPC of 1.7 g·L^-1^ (**Figure 1I**). Only ‘Porter’s Perfection’ had a significant negative linear relationship between TPC and crop density (*P* < 0.0001).

### Juice polyphenol monomers

**Figure 2:**
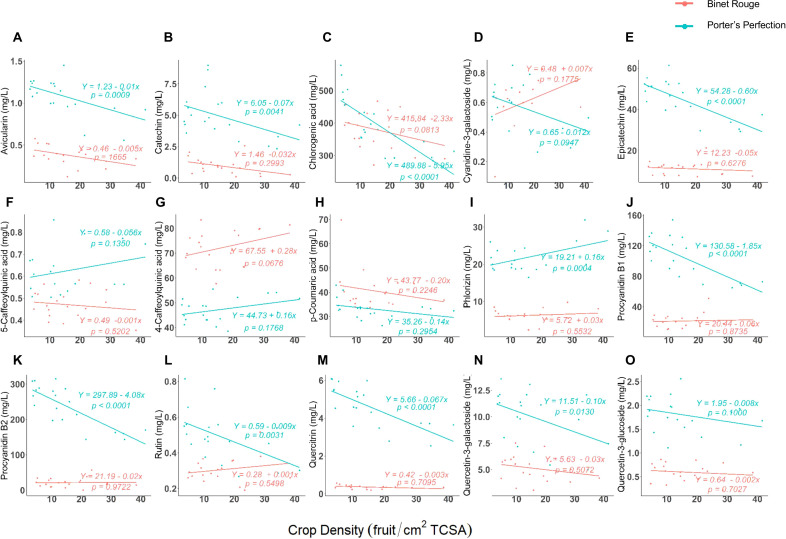
**(A–O)** Regression between crop density [fruit per trunk cross-sectional area (TCSA)] and at-harvest polyphenol compounds from ‘Porter’s Perfection’ and ‘Binet Rouge’ apples harvested from trees subjected to four crop density treatments. Each datapoint represents a composite juice sample for 10 apples per experimental unit.

Ten of the fifteen polyphenol compounds measured in the extracted juice had a significant correlation with crop density for at least one cultivar (**Figure 2**). ‘Porter’s Perfection’ consistently had a significant negative correlation for avicularin, catechin, chlorogenic acid, epicatechin, procyanidin B1, procyanidin B2, quercetin-3-galactocide, quercitrin, and rutin and crop density, while phlorizin which had a positive linear correlation. There were no significant correlations between the polyphenol monomers and crop density for ‘Binet Rouge’. ‘Porter’s Perfection’ generally had greater concentrations of the phenolic compounds than ‘Binet Rogue’. However, 4-caffeoylquinic acid, cyanidine-3-galactoside, and p-coumaric acid (at the greater crop loads) were in greater concentration in ‘Binet Rogue’.

For ‘Porter’s Perfection’, chlorogenic acid, procyanidin B1, and procyanidin B2 had the greatest concentrations (>100 mg·L^-1^), 4-caffoeoylquinic acid, epicatechin, p-coumaric acid, phlorizin, and quercetin-3-galactoside had medium concentrations (10–100 mg·L^-1^), and the remaining compounds had low concentrations (<10 mg·L^-1^). For ‘Binet Rouge’, only chlorogenic acid had a high concentration (>100 mg·L^-1^), 4-caffoeoylquinic acid, epicatechin, p-coumaric acid, procyanidin B1, and procyanidin B2 had medium concentrations (10–100 mg·L^-1^), and the rest of the compounds had low concentrations (<10 mg·L^-1^). Among all the polyphenol compounds measured, chlorogenic acid had the greatest concentration with an average concentration of 399 and 379 mg·L^-1^ for ‘Porter’s Perfection’ and ‘Binet Rouge’, respectively (**Figure 2C**).

There were four proanthocyanidin compounds that were identified in the juice of both cultivars: catechin, epicatechin, procyanidin B1 (catechin-epicatechin dimer), and procyanidin B2 (epicatechin-epicatechin dimer) (**Figures 2B, E, J, K**). Among the procyanidins, catechin had the lowest concentration at 5 and 1 mg·L^-1^ for ‘Porter’s Perfection’ and ‘Binet Rouge’, respectively (**Figure 2B**), whereas procyanidin B2 had the greatest concentrations at 236 mg·L^-1^ and 22 mg·L^-1^ for ‘Porter’s Perfection’ and ‘Binet Rouge’, respectively (**Figure 2K**). Overall, the measured phenolic compounds were more responsive to crop load for ‘Porter’s Perfection’ than ‘Binet Rouge’. Thus, we further analyzed the flesh, peel, and juice for ‘Porter’s Perfection’ over the course of the season.

### Flesh and peel tannin and polyphenols in ‘Porter’s Perfection’

**Figure 3:**
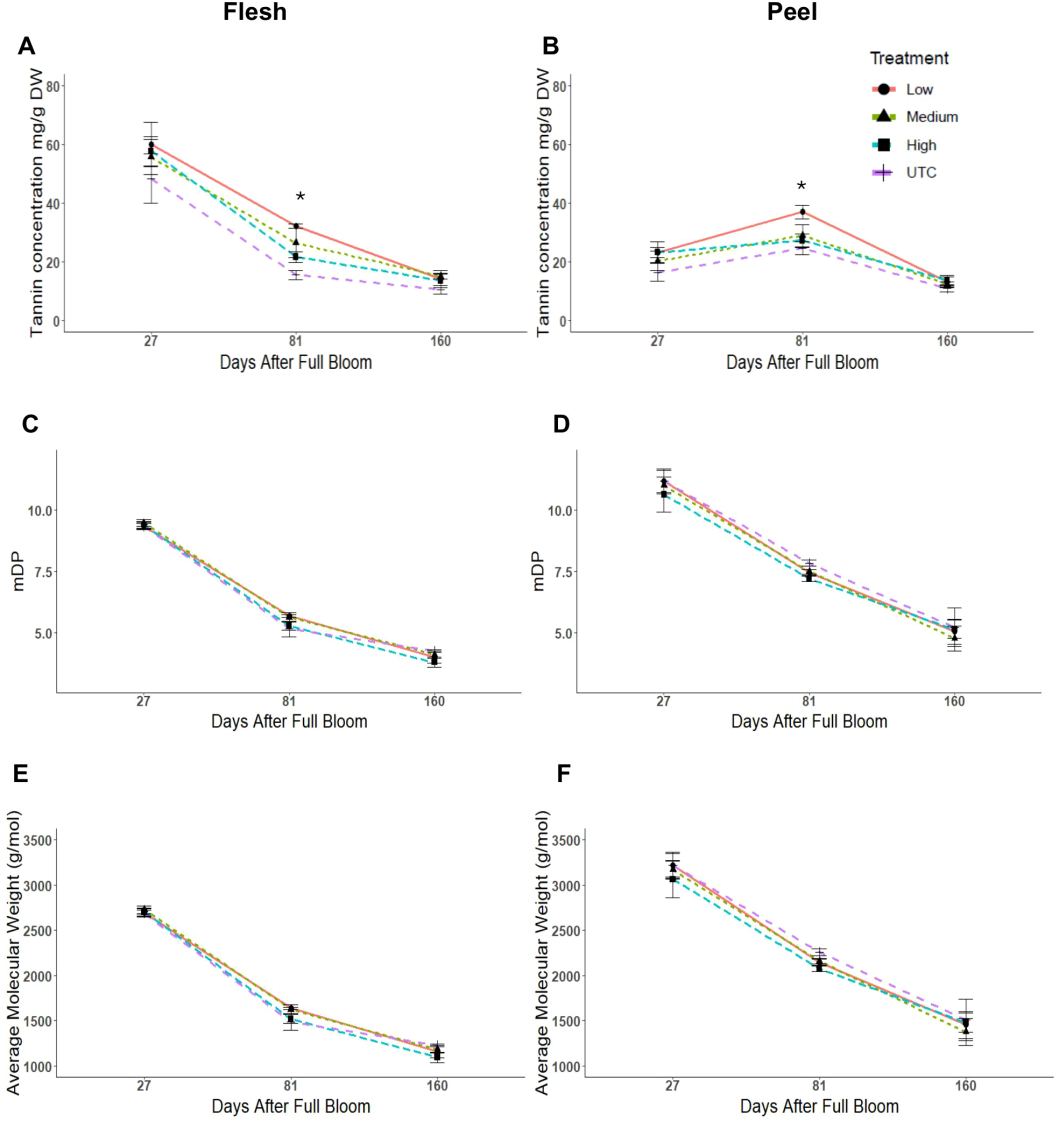
Tannin concentration, mean degree of polymerization (mDP), and average molecular weight of tannins from ‘Porter’s Perfection’/’G.11’ flesh **(A, C, E)** and peel **(B, D, F)** apple tissue harvested at 27, 81, and 160 days after full bloom from trees subjected to Low, Medium, and High crop density treatments (thinned to 5, 10, and 15 fruit/cm^2^ fruit per trunk cross-sectional area, respectively), and an UnThinned Control (UTC). *Signifies mean separation at *P* ≤ 0.05.

‘Porter’s Perfection’ flesh and peel tannin concentrations were analyzed at 27, 81, and 160 DAFB (**Figure 3**). At 27 DAFB, the flesh had an average tannin concentration of 55 mg·g^-1^ of dry weight (DW) tissue which reduced to 24 and then to 13 mg·g^-1^ DW at 81 and 160 DAFB (**Figure 3A**). Peel tannin concentration was lowest at 27 DAFB (22 mg·g^-1^ DW), increased to 29 mg·g^-1^ DW at 81 DAFB, and then reduced to 12 mg·g^-1^ DW at 160 DAFB (**Figure 3B**). At 81 DAFB, there was a significant increase in average tannin concentrations of the Low crop density treatment to 108% and 50% greater than the UTC treatments for the flesh (*P* = 0.0209) and peel (*P* = 0.0032) tissues. While the Low treatments had greater concentrations of tannin in comparison to the UTC at 27 and 160 DAFB, the differences were not statistically significant. Although flesh tannin concentration was twice that of the peel at the first sampling, by 160 DAFB, both the flesh and peel had similar concentrations.

There were no significant differences in the mean degree of polymerization (mDP) and the average molecular weight of tannins among treatments at any time point for peel and flesh tissues (**Figures 3C, D**). The peel tissue maintained a greater level of polymerization than the flesh throughout the growth season decreasing from 11 mDP at 27 DAFB to 5 mDP at 160 DAFB. The flesh tissue average decreased from 9 mDP at 27 DAFB to 4 mDP at 160 DAFB. The peel had greater average molecular weight values in comparison to the flesh tissue throughout the experiment. There was a decrease in molecular weight by ~55% from 27 DAFB to 160 DAFB in both peel and flesh tissues (**Figures 3E, F**).

**Figure 4:**
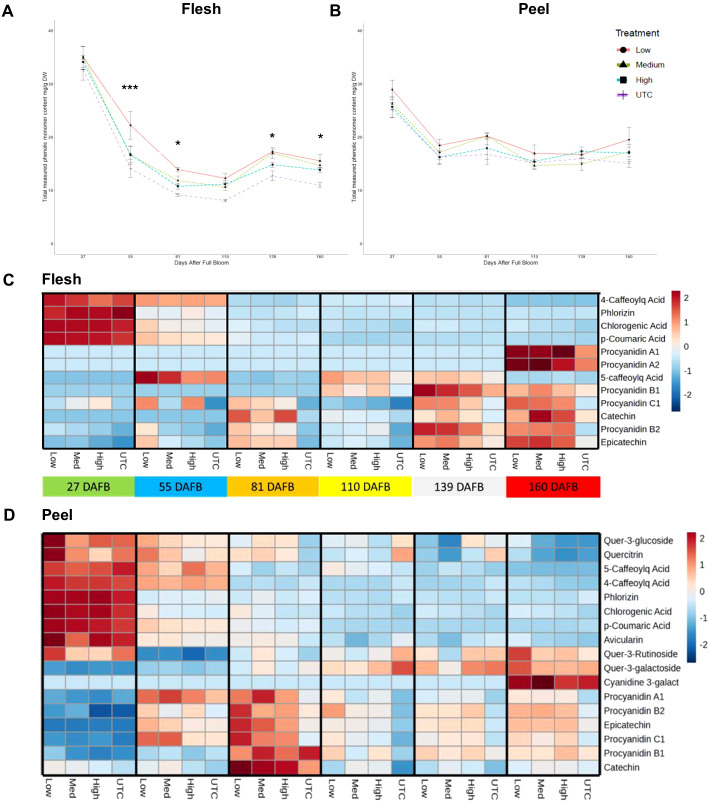
Total measured monomeric phenolic compounds from ‘Porter’s Perfection’/’G.11’ flesh **(A)** and peel **(B)** apple tissue harvested at 27, 81, and 160 days after full bloom (DAFB) from trees subjected to Low, Medium, and High crop density treatments (thinned to 5, 10, and 15 fruit/cm^2^ fruit per trunk cross-sectional area, respectively), and an UnThinned Control (UTC). *, *** signifies mean separation at *P* ≤ 0.05 or 0.001, respectively. The relative [minimum (-2) and maximum values (2)] accumulation patterns of individual polyphenol monomers or oligomers in the flesh **(C)** and peel **(D)** tissue. Cyanidinine-3-galact, Cyanidine-3-galactoside; Caffeoylq Acid, Caffeoylquinic Acid.

Polyphenol compounds were measured at six time points for both peel and flesh tissues of ‘Porter’s Perfection’ at approximately four-week intervals from the implementation of the treatments until harvest (**Figures 4A, B**). On a dry weight basis, both the flesh and peel tissue had the greatest concentrations at 27 DAFB with an average of 34 mg·g^-1^ DW and 27 mg·g^-1^ DW, respectively. There was a 49% and 35% reduction of measured polyphenol concentration from 27 DAFB to 55 DAFB for flesh and peel tissue which was maintained (± 6 mg·g^-1^ DW for flesh and (± 2 mg·g^-1^ DW for flesh) until 160 DAFB. In the peel tissue there were no significant differences among treatments. The flesh tissue maintained a significantly greater polyphenol concentration in the low crop density vs UTC treatments from a 57% increase at 55 DAFB (*P* < 0.0001) to a 41% increase in concentration at 160 DAFB (*P* = 0.0460).

Twelve polyphenol compounds were quantified in the flesh and seventeen were quantified in the peel tissue of ‘Porter’s Perfection’ (**Figures 4C, D**). Procyanidins A1, A2, and C1 were identified in the flesh tissue, while flavonols such as quercetin glycosides were absent; in the peel tissue, procyanidins A1 and C1 were detected, but none of these compounds were present in the juice samples. In both flesh and peel tissues, there was a greater concentration of phenolic acids and phlorizin at 27 DAFB and then a gradual decrease in concentration until 160 DAFB. A similar trend was found in peel tissue for flavonols, such as quercitrin and quercetin-3-rutinoside. In the peel tissue proanthocyanidins accumulated at 55 and 81 DAFB and then decreased until 160 DAFB. In the flesh tissue proanthocyanidins accumulated gradually and reached their peak concentration at the pre-harvest (139 DAFB) and harvest (160 DAFB) stages.

### RNA sequencing and differential gene expression

**Figure 5:**
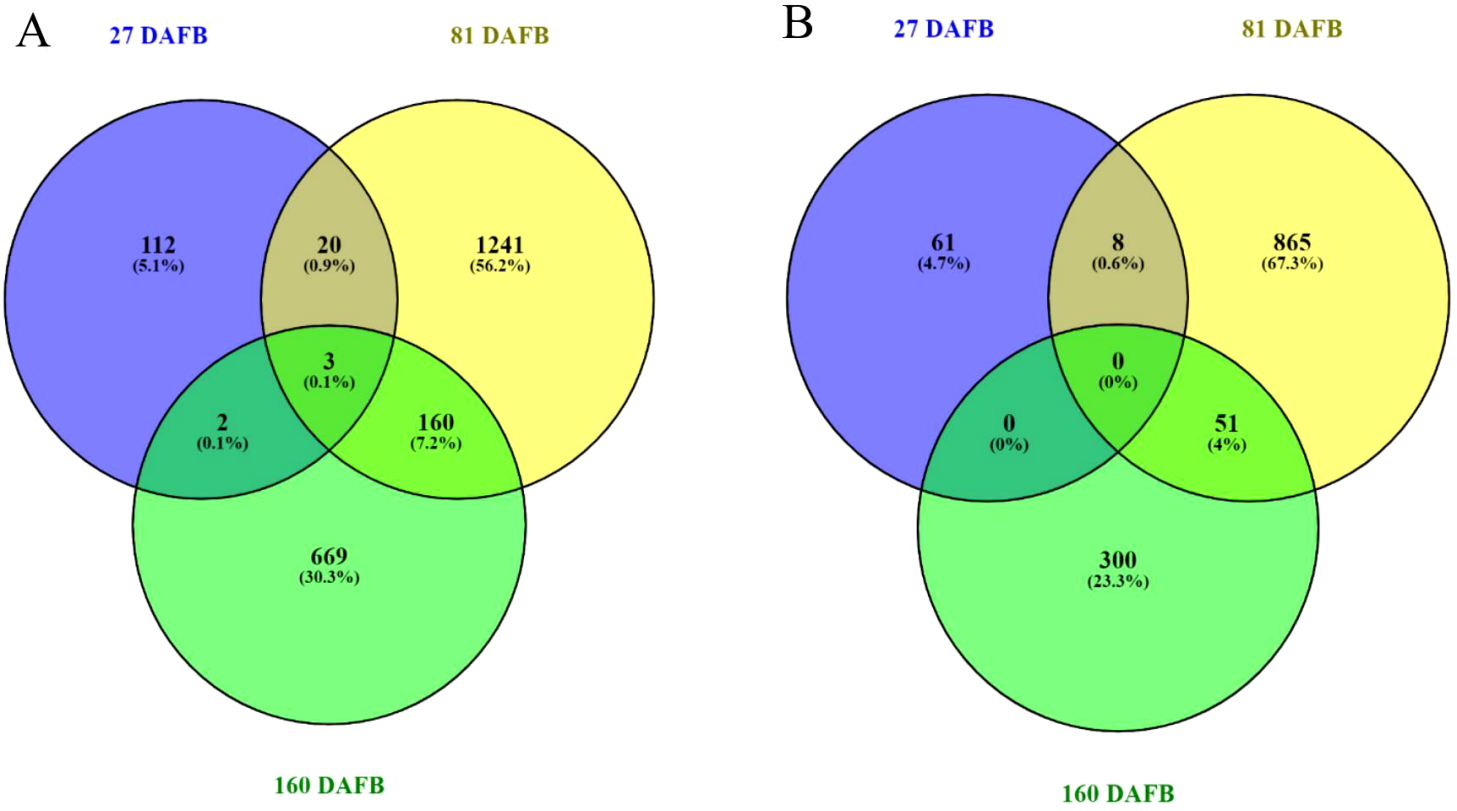
Venn diagrams outline the overlapping and distinct differentially expressed genes between the Low crop density (5 fruit/cm^2^ fruit per trunk cross-sectional area) and UnThinned Control (UTC) treatments at 27, 81, and 160 days after full bloom (DAFB) in the flesh **(A)** and peel **(B)** apple tissue harvested from ‘Porter’s Perfection’/’G.11’ trees.

RNA sequencing was used to identify differential expressed genes (DEGs) between the lowest (5 fruit/TCSA) and the greatest (UTC) crop density treatments for ‘Porter’s Perfection’. Both flesh and peel tissue were analyzed at 27, 81, and 160 DAFB. Comparing the UTC and low crop density treatments there were 2,207 DEGs in the flesh and 1,285 DEGs in the peel tissue across the three sampling time points (**Figure 5**; **Supplementary Table S1**). At 27 DAFB there were 74 downregulated and 63 upregulated DEGs in the flesh tissue; and 36 downregulated and 33 upregulated DEGs in peel tissue. The greatest number of DEGs were found at 81 DAFB with 509 downregulated and 915 upregulated DEGs in the flesh tissue; and 420 downregulated and 504 upregulated DEGs in the peel tissue. At 160 DAFB, there were 476 downregulated and 358 upregulated DEGs in the flesh tissue; and 137 downregulated and 214 upregulated DEGs, respectively. There was more overlap of shared DEGs between 81 and 160 DAFB than between 27 and 81 DAFB for both flesh and peel tissues. There were only 20 and 8 genes shared between 27 and 81 DAFB in the flesh and peel, respectively, whereas there were 160 and 51 genes shared between 81 and 160 DAFB in the flesh and peel, respectively. The flesh tissue had three genes that were differentially expressed at all three sample times, whereas the peel did not have any shared genes throughout the three sample times.

### Gene enrichment analyses

**Figure 6:**
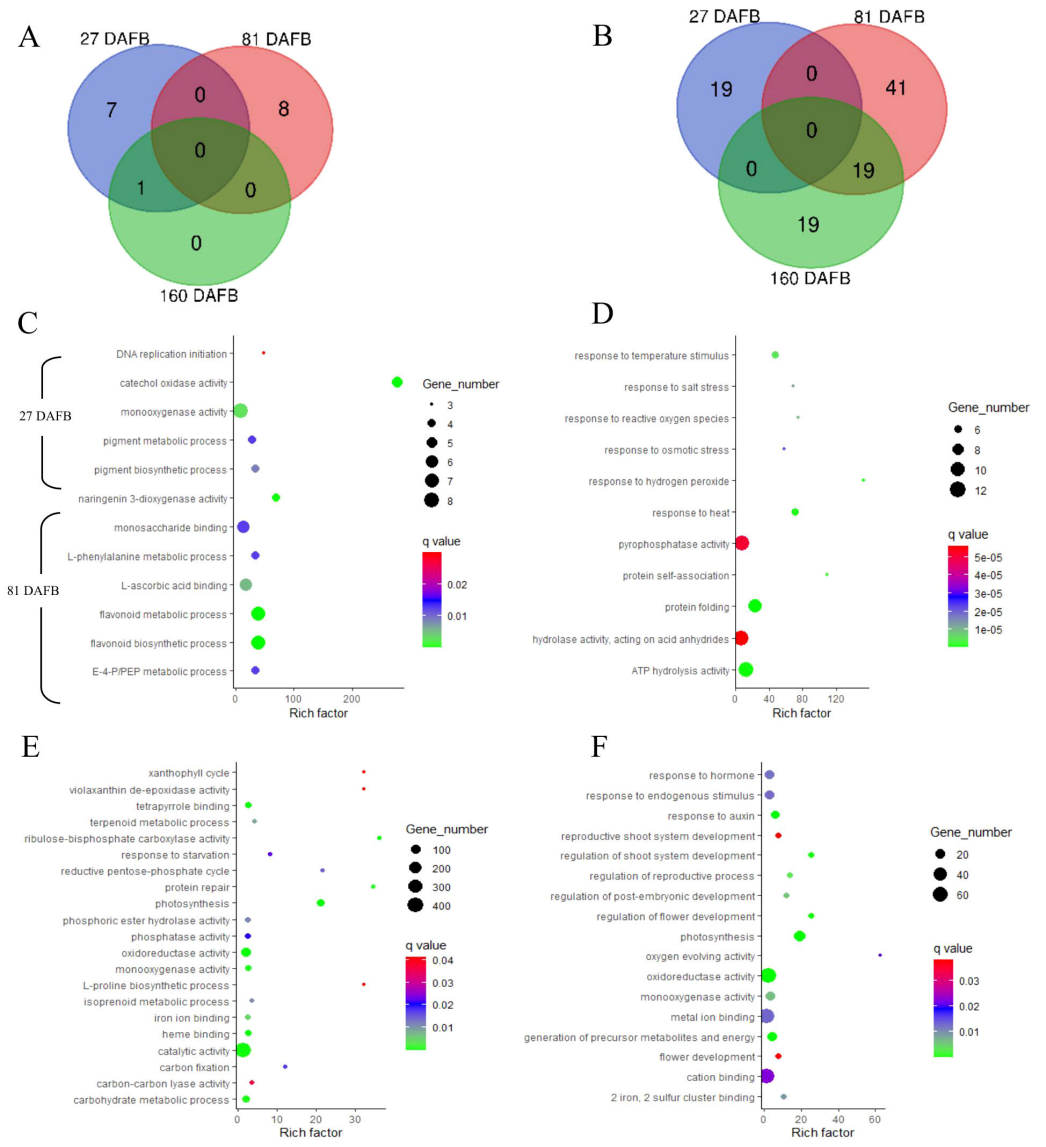
Venn diagrams outline the overlapping and distinct enriched gene ontology (GO) terms between the Low crop density (5 fruit/cm^2^ fruit per trunk cross-sectional area) and UnThinned Control (UTC) treatments that were up **(A)** and downregulated **(B)** at 27, 81, and 160 days after full bloom (DAFB) in the apple flesh tissue harvested from ‘Porter’s Perfection’/’G.11’ trees. Relevant enriched GO terms that upregulated at 27 and 81 DAFB are presented in **(C)** (there were minimal enriched GO terms that were upregulated at 160 DAFB). Relevant enriched GO terms downregulated at 27 DAFB, 81 DAFB, and 160 DAFB are presented in **(D–F)**, respectively. Rich factor percent is the ratio between the differentially expressed genes annotated and the total of all genes annotated in a pathway. The q-value is the minimum false discovery rate at which an observed score is deemed significant. E-4-P/PEP, erythrose 4-phosphate/phosphoenolpyruvate family amino acid metabolic process.

**Figure 7:**
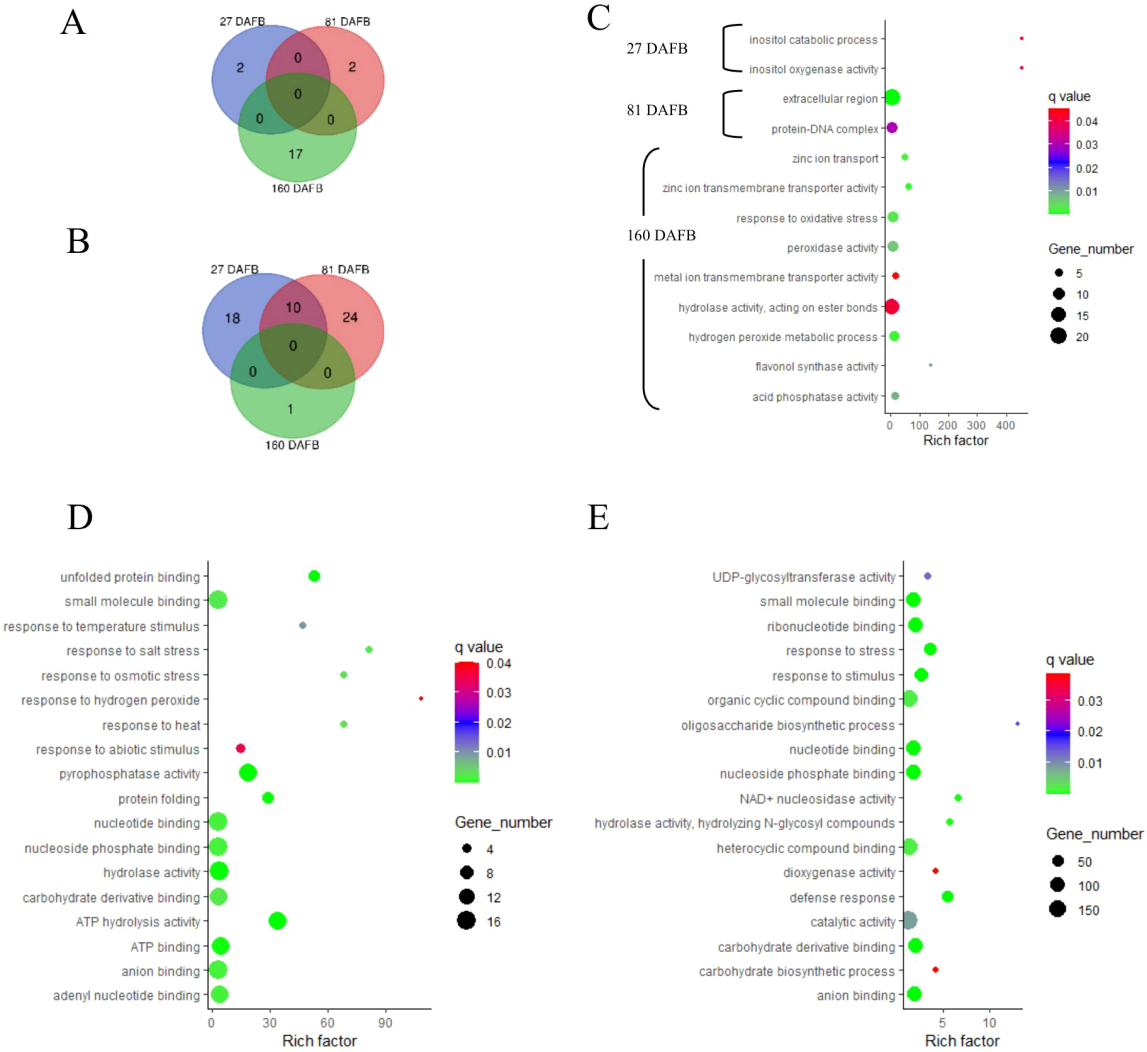
Venn diagrams outline the overlapping and distinct enriched gene ontology (GO) terms between the Low crop density (5 fruit/cm^2^ fruit per trunk cross-sectional area) and UnThinned Control (UTC) treatments that were up **(A)** and downregulated **(B)** at 27, 81, and 160 days after full bloom (DAFB) in the apple peel tissue harvested from ‘Porter’s Perfection’/’G.11’ trees. Relevant enriched GO terms upregulated at 27, 81, and 160 DAFB are presented in **(C)** Select enriched GO terms downregulated at 27 DAFB and 81 DAFB are presented in **(D, E)**, respectively (there were minimal enriched GO terms that were downregulated at 160 DAFB). Rich factor percent is the ratio between the differentially expressed genes annotated and the total of all genes annotated in a pathway. The q-value is the minimum false discovery rate at which an observed score is deemed significant.

Enriched gene ontology (GO) terms (*P* ≤ 0.05) are presented in **Figures 6** and **7** for flesh and peel, respectively, and a detailed list is provided in **Supplementary Table S2**. There were more enriched GO terms that were downregulated for the low crop density treatment for both flesh and peel tissues. Fruit samples taken at 81 DAFB showed the maximum number of DEGs and enriched GO terms for both flesh and peel tissue (**Figures 6**, **7**). In flesh tissue, there were a total of 16 GO terms that were upregulated, whereas there were 98 GO terms that were downregulated. There were 19 downregulated GO terms shared between 81 DAFB and 160 DAFB in flesh (**Figures 6A, B**). Among the GO terms upregulated at 81 DAFB in flesh, biological processes such as “L-phenylalanine metabolic process”, “flavonoid biosynthetic process”, “flavonoid metabolic process”, and molecular functions such as “naringenin 3-dioxygenase activity” are directly involved in the phenylpropanoid pathway synthesis and regulation (**Figure 6C**). Among the upregulated GO terms at 81 DAFB in flesh, “flavonoid biosynthetic and metabolic processes” had the greatest gene number (n = 7) within their respective GO terms (**Figure 6C**).

Among the downregulated GO terms at 27 DAFB in flesh, there was a downregulation of stress response genes including those involved in salt stress, reactive oxygen species, osmotic stress, heat, and hydrogen peroxide in the Low crop density treatment, indicating increased stress response for the UTC treatment (**Figure 6D**). Molecular functions such as “pyrophosphatase activity”, “hydrolase activity”, and “ATP hydrolase activity” had greater number of genes (10-12) within their GO terms than others (**Figure 6D**). Among the downregulated GO terms at 81 and 160 DAFB in flesh, there were multiple shared GO terms relating to photosynthesis including, but not limited to, photosystems 1 and 2, thylakoid membrane, generation of precursor metabolites and energy, photosynthetic membranes (**Figures 6E, F**; **Supplementary Table S2**). At 81 DAFB, there was also a downregulation of the carbohydrate metabolic process and Rubisco (ribulose-bisphosphate carboxylase activity). At 160 DAFB, the GO terms had a marked downregulation of hormone function, including “response to auxin”.

The peel tissue had two upregulated GO terms at 27 and 81 DAFB and 17 upregulated GO terms at 160 DAFB (**Figure 7A**). There were 28 and 34 downregulated genes at 27 and 82 DAFB, with 10 GO terms shared between the sample dates. At 160 DAFB, there was only one downregulated GO term (**Figure 7B**). Flavonol synthase activity and GO terms related to zinc ion transport were upregulated at 160 DAFB in the peel tissue (**Figure 7C**). Among the downregulated GO terms at 27 and 81 DAFB in peel, response to different stresses such as salt, osmotic, hydrogen peroxide, and abiotic stimuli featured prominently in both the growth stages (**Figures 7D, E**). Interestingly, the term “carbohydrate derivative binding” was also downregulated at both 27 and 81 DAFB (**Figures 7D, E**). Additionally, there were several GO terms involved in molecular function “binding”, such as “nucleotide binding”, “nucleoside phosphate binding”, “ATP binding”, and “adenyl nucleotide binding” that were downregulated at 27 DAFB in peel tissue, and many of these GO terms have 12–16 individual genes within their GO terms (**Figure 7D**). At 81 DAFB, there was a downregulation of the “carbohydrate biosynthetic process”, as well as “UDP-glycosyltransferase”, both GO terms being very essential in the phenylpropanoid pathway (**Figure 7E**).

### Phenylpropanoid pathway genes

**Figure 8:**
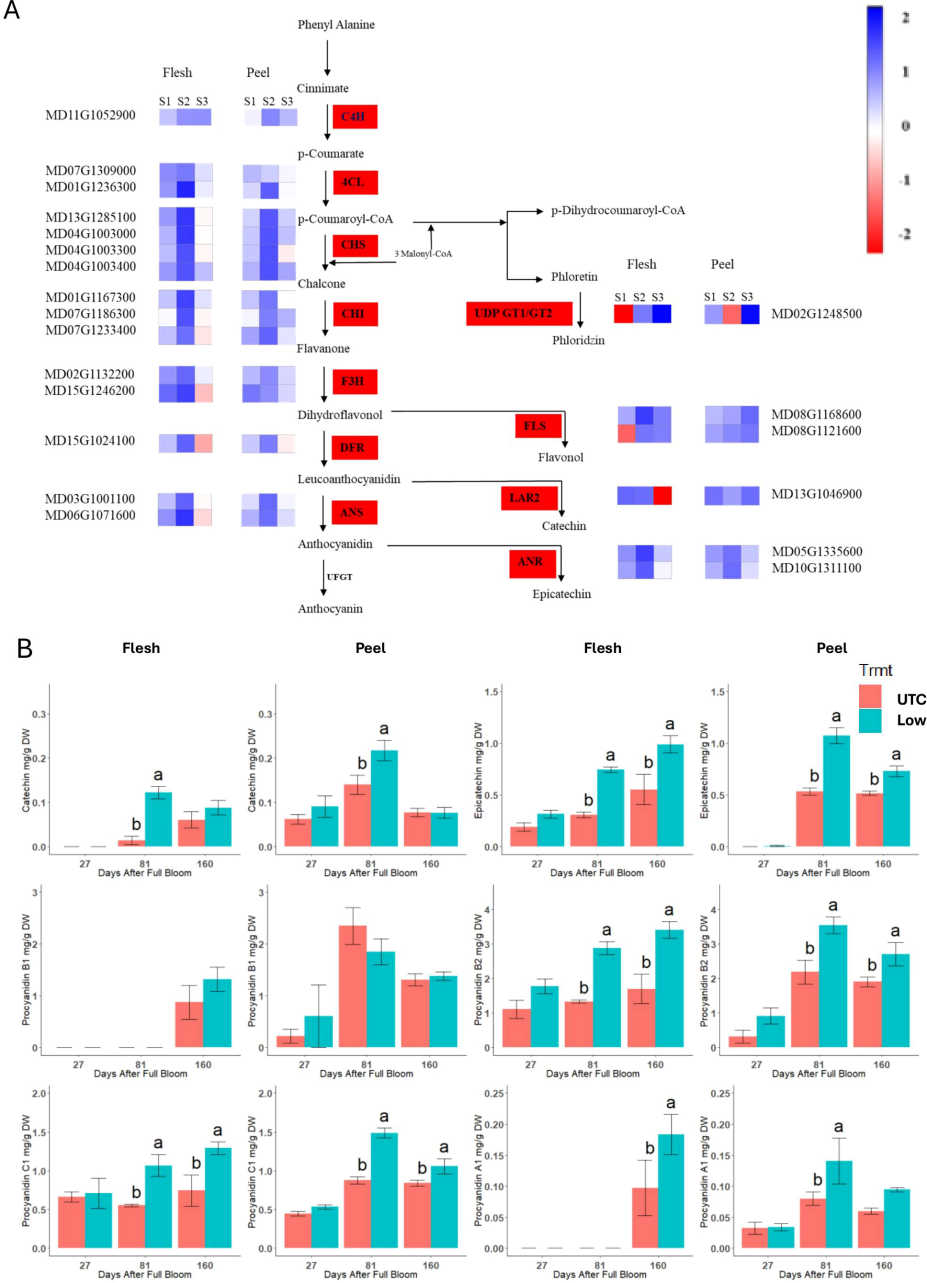
**(A)** Differentially expressed genes in the phenylpropanoid pathway between the Low crop density (5 fruit/cm^2^ fruit per trunk cross-sectional area) and UnThinned Control (UTC) treatments in apple flesh and peel tissue from ‘Porter’s Perfection’ trees. Only differentially expressed genes with at least a two-fold difference in expression and adjusted *P* ≤ 0.05 in any one of the developmental stages are shown. Individual polyphenol monomers comparing the UTC and the low crop density treatment for flesh and peel tissue of the cultivar ‘Porter’s Perfection’. Sample points S1, S2, and S3 represent 27, 81, and 160 Days After Full Bloom, respectively. **(B)** Means within each time point followed by different lowercase letters are significantly different based on the Tukey’s HSD means comparison at α = 0.05. PAL, phenyl ammonia lyase; C4H, cinnamate-4-hydroxymate; 4CL, 4-coumarate: coenzyme A ligase; CHS, chalcone synthase; CHI, chalcone isomerase; F3H, Flavanone 3-hydroxylase; DFR, dihydroflavonol 4-reductase; ANS, anthocyanidin reductase; UFGT, UDP-glucose flavonoid 3-O-glucosyl transferase; FLS, flavonol synthase; LAR1/2, Leucoanthocyanidin reductase; GT1/2, glycosyl transferases.

The phenylpropanoid pathway genes presented in **Figure 8A** were upregulated at 81 DAFB in the low crop density treatments. A threshold of at least a 2-fold down- or upregulation with a *P* ≤ 0.05 was used to select genes for further scrutiny. There was no significant upregulation at 27 DAFB and 160 DAFB for most of the genes. In general, the flesh tissue had a greater upregulation of phenylpropanoid pathway genes at 81 DAFB than the peel tissue. Even among genes that had a 2-fold upregulation, some were up-regulated to a significantly greater degree. The 4-coumarate CoA ligase gene *MD01G1236300* exhibited a 6.25-fold increase in expression in the flesh tissue at 81 DAFB (**Figure 8A**). Similarly, two of the chalcone synthase genes *MD13G1285100* and *MD04G1003000*, and a flavonol synthase gene *MD08G1168600* exhibited a 4-fold upregulation in the flesh tissue at 81 DAFB. Anthocyanidin synthase (*MD06G1071600*) and anthocyanidin reductase (*MD05G1335600*) genes are the penultimate and the final steps, respectively, in the production of epicatechin, and both genes exhibited significant upregulation (4.75 and 3.85-fold, respectively) at 81 DAFB in the flesh tissue. Concentrations of the procyanidin monomers and dimers were greater in the low crop density treatment than in the UTC (**Figure 8B**). Except for procyanidin B1, all the compounds had a significant increase at 81 DAFB mirroring the increase in the expression of key genes at 81 DAFB. The differences in proanthocyanidin concentrations between the Low crop density and the UTC treatments were sustained throughout to 160 DAFB for all the procyanidins except for catechin, procyanidin B1, and procyanidin A1 in the peel tissue.

### Transcription factor DEGs

**Figure 9:**
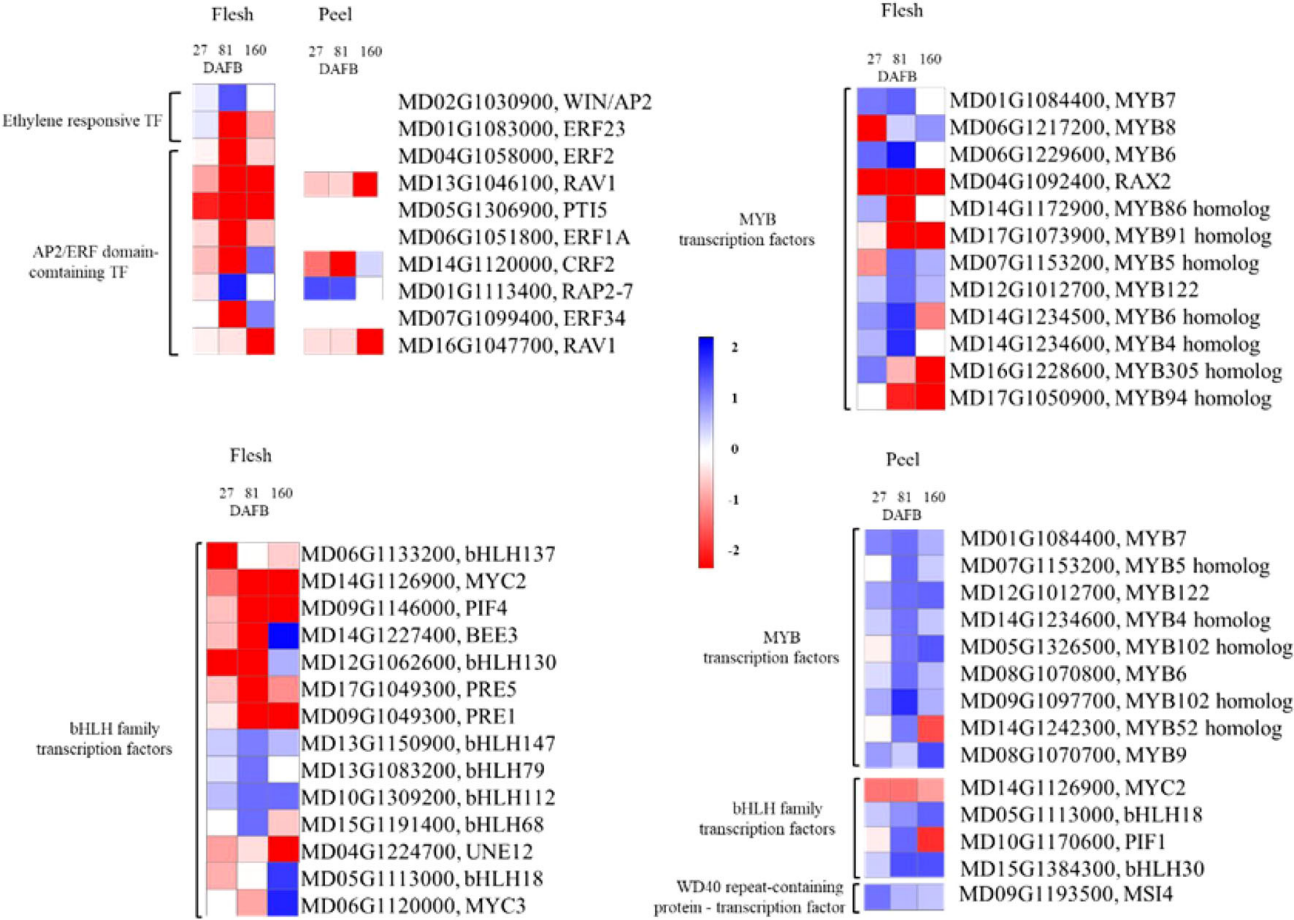
The differentially expressed genes in the ethylene-responsive transcription factors (ERF) and MYB-bHLH-WD40 complex signaling pathway comparing the Low crop density (5 fruit/cm^2^ fruit per trunk cross-sectional area) and UnThinned Control (UTC) treatments in apple flesh and peel tissue from ‘Porter’s Perfection’ at 27, 81, and 160 days after full bloom (DAFB). Only differentially expressed genes with at least a two-fold difference in expression and a *P* ≤ 0.05 in at least one of the developmental stages are shown. MYB, myeloblastosis viral oncogene homolog; bHLH, basic Helix Loop Helix; WD, beta-transducin repeat.

The ethylene responsive factors (ERF) have been found to be involved in regulation of various primary and secondary metabolism pathways, including the phenylpropanoid pathway. There was a total of 10 and 4 ERF DEGs in the flesh and peel tissues, respectively (**Figure 9**). At 27 DAFB, there were no differences in expression for the ERF genes. At 81 and 160 DAFB, the ERFs were downregulated in the low crop density treatment with a notable exception in “AP2 like ERF” (*MD01G1113400*), which witnessed a significant upregulation at 81 DAFB for both flesh (7-fold) and peel (3-fold).

The MYB-bHLH-WD40 complex has previously been found to control the production of different polyphenol compounds including proanthocyanidins. There were twelve and nine MYB DEGs identified in the flesh and peel tissues, respectively (**Figure 9**). There were fourteen bHLH DEGs in the flesh tissue and four in the peel tissue. There was only one WD-40 DEG identified in the peel tissue (**Figure 9**). The flesh tissue in general had more MYB-bHLH-WD40 DEG’s than the peel tissue.

In the flesh tissue, there was one significant MYB DEGs downregulated at 27 DAFB (*MD06G1217200*). MYB DEGs at 160 DAFB had four downregulated MYB TFs in the low crop density treatment (*MD04G1092400, MD17G1073900, MD16G1228600*, and *MD17G1050900*) (**Figure 9**). The majority of the MYB TFs were differentially expressed at 81 DAFB, with four upregulated (*MD07G1153200*, *MD12G1012700*, *MD14G1234500*, and *MD14G1234600*) and three downregulated (*MD04G1092400*, *MD14G1172900*, and *MD17G1073900*) in the low crop density treatment. The flesh bHLH TFs had a similar trend as their MYB counterparts. While there was no significant effect of the treatments on MYB genes at 27 DAFB, there were six bHLH genes downregulated and four upregulated at 81 DAFB, whereas there were four genes upregulated and three downregulated at 160 DAFB. Notably, there was one bHLH gene (*MD14G1227400*) that had an ~3.5-fold downregulation at 81 DAFB and an ~17-fold upregulation at 160 DAFB.

In the peel tissue, there were no MYB-bHLH-WD40 DEGs that were significantly different from the UTC at 27 DAFB, whereas there was a general trend of upregulation for these genes at 81 and 160 DAFB (**Figure 9**). Notably, there was one MYB TF (*MD09G1097700*) that had a 5-fold upregulation at 81 DAFB. One bHLH TF (*MD15G1384300*) had a 3-fold upregulation at both 81 and 160 DAFB.

## Discussion

### Polyphenol concentrations are negatively correlated with crop density

At harvest analysis of juice TPC illustrated a negative correlation of crop density with TPC for both cultivars, albeit significant only for ‘Porter’s Perfection’. This correlation is corroborated with evidence from Karl and Peck (2022), who observed that lower crop densities in one season in the French bittersweet apple ‘Medaille d’Or’ increased polyphenol concentrations. Zakalik et al. (2023) also found over a 3-year study that increasing crop density decreased TPC for all the seven cider apple cultivars studied, including ‘Binet Rouge’. Most apple fruit polyphenols (individual polyphenol compounds and tannins) appear to be produced in the initial cell-division stage of fruit growth until about 30 DAFB and then reduce in concentrations until harvest (Guyot et al., 2003; Renard et al., 2007; Plotkowski and Cline, 2021a).

### Carbohydrate availability has a role to play in accumulation of polyphenols in cider apple

We hypothesize that carbohydrate availability during the cell division phase of fruit growth (up to 30 DAFB) is the primary controlling factor of polyphenol biosynthesis in apples. Primary and secondary metabolism is dependent on carbohydrate availability during the crucial cell division phase from 1 to 30 DAFB where carbohydrates are necessary for cell division, growth, and function (Lakso et al., 1989). In this period of cell division in fruit, existing carbohydrate reserves preferentially assist shoot growth and extension rather than invest in fruit sinks, hence the fruit are dependent on localized carbohydrates produced from spur leaves adjacent to the fruit (Bepete and Lakso, 1998). An increased crop density would reduce primary and secondary metabolite production and accumulation in individual fruit due to non-availability of enough carbohydrate to satisfy sink demands. Anthony et al. (2023) and Bell and Henschke (2005) identified that low crop density in peach and wine grape, respectively, enhanced carbon availability early in the season to ensure consistent synthesis of flavonoids, such as catechin and epicatechin, and the fruit maintained those high levels of polyphenols until harvest in comparison to untreated controls.

### RNA Sequencing, gene enrichment analysis, and differential gene expression

The Low crop density treatment was compared with UTC at three timepoints: 27, 81, and 160 DAFB for ‘Porter’s Perfection’ peel and flesh tissues. The 27 DAFB measurements effectively served as controls because samples were taken only a few days after implementation of the crop density treatments and, as expected, there were not many significant differences. The 81 DAFB timepoint had the most differences in gene expression with 1,241 DEGs in the flesh and 865 DEGs in the peel tissue. In the gene enrichment analyses, there was an upregulation of the flavonoid metabolism and flavonoid biosynthesis pathway in the low crop density treatment in comparison to the UTC. This was further probed by examining key polyphenol pathway genes in the cider cultivar ‘Porter’s Perfection,’ which showed a strong effect of reduced crop density on upregulation of polyphenol pathway genes, especially at 81 DAFB. Specifically, in the Low crop density treatment, there was an upregulation in the gene encoding anthocyanidin reductase (ANR), which catalyzes the synthesis of epicatechin (Takos et al., 2006a; Henry-Kirk et al., 2012). Furthermore, there was also an upregulation of the L-phenylalanine metabolic process in the Low crop density treatment, which is a key substrate needed for phenylalanine ammonia lyase to biosynthesize phenylpropanoids (Fanyuk et al., 2022).

At 81 DAFB, the Low crop density treatment also decreased carbohydrate production via the downregulation of photosynthesis, photosystems, and rubisco (a key enzyme involved in regulating carbon assimilation rates) activity. Lower crop densities have been shown to reduce sink demand and leaf carbon assimilation for apple trees (Palmer et al., 1997; Wünsche et al., 2000; Yang et al., 2021), thus downregulating photosynthesis and carbohydrate accumulation. We also observed lower levels of glyceraldehyde-3-phosphate dehydrogenase, which is involved in the first step of converting glyceraldehyde-3-phosphate (G-3-P) to ribulose bisphosphate (RuBP), another substrate for carbon fixation (Sharkey, 1985). In other words, once carbon sufficiency is reached during the initial phase of fruit growth in the low crop density treatment, there is a downregulation of carbohydrate production processes due to a lower sink demand, thus, there is an effective adjustment of carbohydrate status based on crop density in order to meet the sink demand (Palmer et al., 1997; Wünsche et al., 2000; Yang et al., 2021).

### Pre-harvest and harvest characteristics

At harvest, the yield of both ‘Porter’s Perfection’ and ‘Binet Rouge’ had a strong positive linear correlation with increase in crop density, as also observed by Zakalik et al. (2023) and Plotkowski and Cline (2021b). While this is generally the case for the “on year” yields, the following year’s return bloom had a strong negative correlation with increase in crop density in these studies. This tendency of reduced return bloom with increasing crop density in the previous year is well established in the literature (Pellerin et al., 2011; Robinson and Watkins, 2003). In fact, trees did not have a single return bloom cluster and were pushed into complete bienniality at any crop density equal to or greater than 28.4 fruit/cm^2^ TCSA for ‘Porter’s Perfection’ and 27.8 fruit/cm^2^ TCSA for ‘Binet Rouge’. Zakalik et al. (2023) observed over three years that a crop density greater than 21.2 fruit/cm^2^ did not have return bloom the next year for ‘Binet Rouge’. The variation in the crop density versus return bloom observed between studies could be attributed to variability in weather conditions, tree spacing and training system, pre-experiment crop density, and/or length of the experiment. Even if the ‘on-year’ yields are greater with increased crop density, cumulative yields over multiple years have shown that increase in crop density resulted in decreased yields in the long-term (Plotkowski and Cline, 2021b; Zakalik et al., 2023).

### Crop density significantly influences polyphenol production in only high polyphenol cultivars

Studies with low polyphenol fresh-market or juice apple cultivars have found minimal to no effects of crop density on polyphenol content (Awad et al., 2001; Peck et al., 2016), whereas studies with high TPC cider cultivars usually responded to increased crop densities with lower TPC (Zakalik et al., 2023; Karl and Peck, 2022). While we were able to observe differences in polyphenol content between the UTC and low crop density treatment in ‘Porter’s Perfection’, we did not observe those differences in ‘Binet Rouge’. There seems to be a cultivar-dependent standard level of polyphenol production in apples irrespective of crop density until a certain threshold, above which a source-sink relationship comes into play to regulate polyphenol development according to crop density. Functional research into the genes especially TFs controlling polyphenol production is necessary to further elucidate the mechanisms of action of polyphenol regulation in cider apples.

### Unique accumulation trends of phloridzin and proanthocyanidins in flesh and peel tissues

Due to significant treatment differences in the cultivar ‘Porter’s Perfection’, we analyzed the trends of polyphenol accumulation throughout the growing season in both flesh and peel tissues. Our research supports previous reports that most polyphenols (individual polyphenol compounds and tannins) are produced in the initial cell-division stage of fruit growth until about 30 DAFB; however, there are some polyphenol compounds that do not follow this accumulation pattern, such as anthocyanins which are synthesized in large quantities in the peel as the fruit ripens (Takos et al., 2006b). Additionally, there was an increase in accumulation of procyanidin monomers and oligomers throughout the growing season and at pre-harvest (139 DAFB) and harvest (160 DAFB) stages for ‘Porter’s Perfection’ flesh tissue. This trend was also observed by Renard et al. (2007) who studied the French cider cultivar ‘Kermerrien’. The accumulation in proanthocyanidin at pre-harvest and harvest stages could be due to the breakdown of larger tannin compounds that decreased in concentration from the first date of measurement until harvest (Renard et al., 2007).

Takos et al. (2006b), Renard et al. (2007), and Henry-Kirk et al. (2012) reported the presence of catechin, epicatechin, and the procyanidins B1, B2, C1 in flesh and peel tissue. In our study, procyanidin A1 (epicatechin-catechin) was found in both flesh and peel, as well as procyanidin A2 (dimeric epicatechin) in the flesh tissue. In the flesh tissue, both procyanidin A1 and A2 were present only at harvest. Strong radical scavenging activity of procyanidin B1 and B2 may be converting them to procyanidin A1 and A2 after utilizing the C-2 hydrogen unit in addition to the o-dihydroxyl structure to neutralize the free radical (Kondo et al., 2000). Our research also confirmed previous reports on polyphenols present only in the peel such as quercetin glycosides and anthocyanins such as cyanidin glycosides (Takos et al., 2006b; Ban et al., 2007). Other compounds such as phenolic acids, proanthocyanidins, and dihydrochalcones are present in both peel and flesh, albeit in different concentrations (Renard et al., 2007; Henry-Kirk et al., 2012).

### Tannin accumulation trends in ‘Porter’s Perfection’

The flesh had greater tannin concentrations than the peel with the maximum accumulation at 27 DAFB and slowly decreased until harvest (160 DAFB). Similarly, mDP decreased from ~9 to 4 units at harvest for both peel and flesh tissues, with the peel tissue exhibiting slightly greater mDP values throughout. Similar trends were observed for the apple cultivar ‘Kermerrien’ (Renard et al., 2007) and in multiple grape cultivars where proanthocyanidin content and mDP decreased to about 50–150 subunits post veraison (Downey et al., 2003; Obreque-Slier et al., 2010). This decreasing mDP trend is not universal. For example, proanthocyanidin monomers polymerized and increased in mDP for the remainder of the growing season for the French cider cultivar ‘Avrolles’ (Renard et al., 2007). Cultivar differences seem to play a major role in the mDP of proanthocyanidins and could explain the increase in mDP in the cultivar ‘Avrolles’ (Guyot et al., 2003). The cultivar differences are influenced by a strong genetic component linked to most polyphenol compounds, which suggests a considerable part of genetic variability in the expression of these traits (Verdu et al., 2014).

### Tannins breakdown into proanthocyanidin monomers and oligomers during the growing season

Tannin concentration appears to be at its maximum early in the season (27 DAFB), which was also observed by other researchers (Renard et al., 2007; Henry-Kirk et al., 2012). At the mid-point of 81 DAFB, we were able to see a significantly greater tannin concentration in the low crop density versus the unthinned control, whereas there were no treatment differences at harvest. However, individual procyanidin monomers and oligomers followed a different trend of gradual accumulation throughout the growing season, especially in the flesh tissue, with a peak accumulation at the pre-harvest and harvest stages. At 81 DAFB, there was a marked upregulation of polyphenol pathway genes for the low crop density treatment which corresponded with an increase in procyanidin concentration; however, the differential gene expression tapered off at 160 DAFB, while the differences in procyanidin content remained at harvest. A breakdown of tannins into procyanidin monomers and oligomers at pre-harvest and harvest stages might be causing the greater procyanidin content in low crop density versus the UTC treatment at harvest. Carbon isotope or green fluorescent protein labelling of procyanidin monomers catechin and epicatechin would provide additional insights into understanding the polymerization and breakdown patterns of phenolics throughout the growing season.

### Transcription factors involved in regulation of the phenylpropanoid pathway

The stress applied through a large crop density in this experiment provided additional understanding the molecular regulation of different aspects of the phenylpropanoid pathway. Specifically, the MYB-bHLH-WD40 and ERF TFs are known to regulate proanthocyanidin production (Xu et al., 2015; Zhang et al., 2018; Li et al., 2020; An et al., 2021). Since the variation in phenylpropanoid levels among treatments was only found in proanthocyanidins, we identified ten differentially expressed ERF genes in the flesh and four in the peel. Eight out of the ten ERF genes were downregulated in the low crop density treatment except for the *MdWIN/AP2* and *MdRAP2–7* genes. Specifically, *MdRAV1* was highly downregulated in the low crop density treatment, indicating a less suppressive effect on proanthocyanidin synthesis as compared to the UTC treatment. MdRAV1 was found to bind to the promoter of *MdANR2* (anthocyanidin reductase), inhibiting its activity (Li et al., 2020). Other studies have indicated that MdERF1A, MdERF1B, and MdERF23 also play a role in in procyanidin regulation (Zhang et al., 2018; Li et al., 2020). We have identified eight novel ERF genes in the flesh and three in the peel that are potentially involved in proanthocyanidin synthesis. We also identified 26 MYB and bHLH DEGs in the flesh and 14 MYB, bHLH, and WD40 DEGs in the peel that could potentially be involved in proanthocyanidin synthesis. Many of these MYB and bHLH genes can bind to the promoters of key genes in the phenylpropanoid pathway such as those encoding phenyl ammonia lyase, chalcone synthase, flavonol synthase, leucoanthocyanidin reductase, and anthocyanidin reductase. Essentially, they act as regulators for these key phenylpropanoid pathway genes. Hence, the functional characterization of these identified novel MYB, bHLH, and ERF genes will help to uncover new regulatory mechanisms for proanthocyanidin production in cider apples.

## Conclusions

This study provides an overview of the molecular regulation of carbohydrate and polyphenol metabolism in cider apples subject to different crop density treatments. Crop density had an inverse relationship with all measured fruit and juice quality variables. Reduced crop density enhanced fruit juice characteristics such as SSC, TA, and TPC. Early season carbohydrate availability during the cell division phase (1–30 DAFB) of fruit growth is hypothesized to be the primary driver of polyphenol production in cider apples. Reduced crop density enhanced the expression of secondary metabolite pathway genes such as the one encoding the L-phenylalanine ammonia lyase, which resulted in an increase in polyphenols and specifically, proanthocyanidin building blocks such as epicatechin through upregulation of the anthocyanidin reductase activity. This study found tannin polymerization and potential breakdown patterns in cider apples that should be validated with carbon isotope studies. Also, key TFs (MYB, bHLH, and ERF) that could be involved in regulating proanthocyanidin production in cider apples have been identified and further functional analysis of these genes would help to uncover regulatory mechanisms of proanthocyanidin production in cider apples.

## Data Availability

The data presented in the study are deposited in the NCBI BioProject database under the accession number PRJNA1004866, and supplementary information in the Cornell E-Commons database https://doi.org/10.7298/kksr-7928.
