# Supplementary material for: Reduced apple crop density enhances total polyphenol accumulation via upregulation of anthocyanidin reductase and other phenylpropanoid pathway genes
Source: Front Plant Sci. 2025 Jun 9;16:1591292. doi: 10.3389/fpls.2025.1591292 (PMC12183023; doi:10.3389/fpls.2025.1591292)
Supplement: Supplementary file 1 [file DataSheet1.docx]

Supplementary Tables and Figures

Supplementary Tables 1 and 2 can be found at <https://doi.org/10.7298/kksr-7928>

**A**

**B**


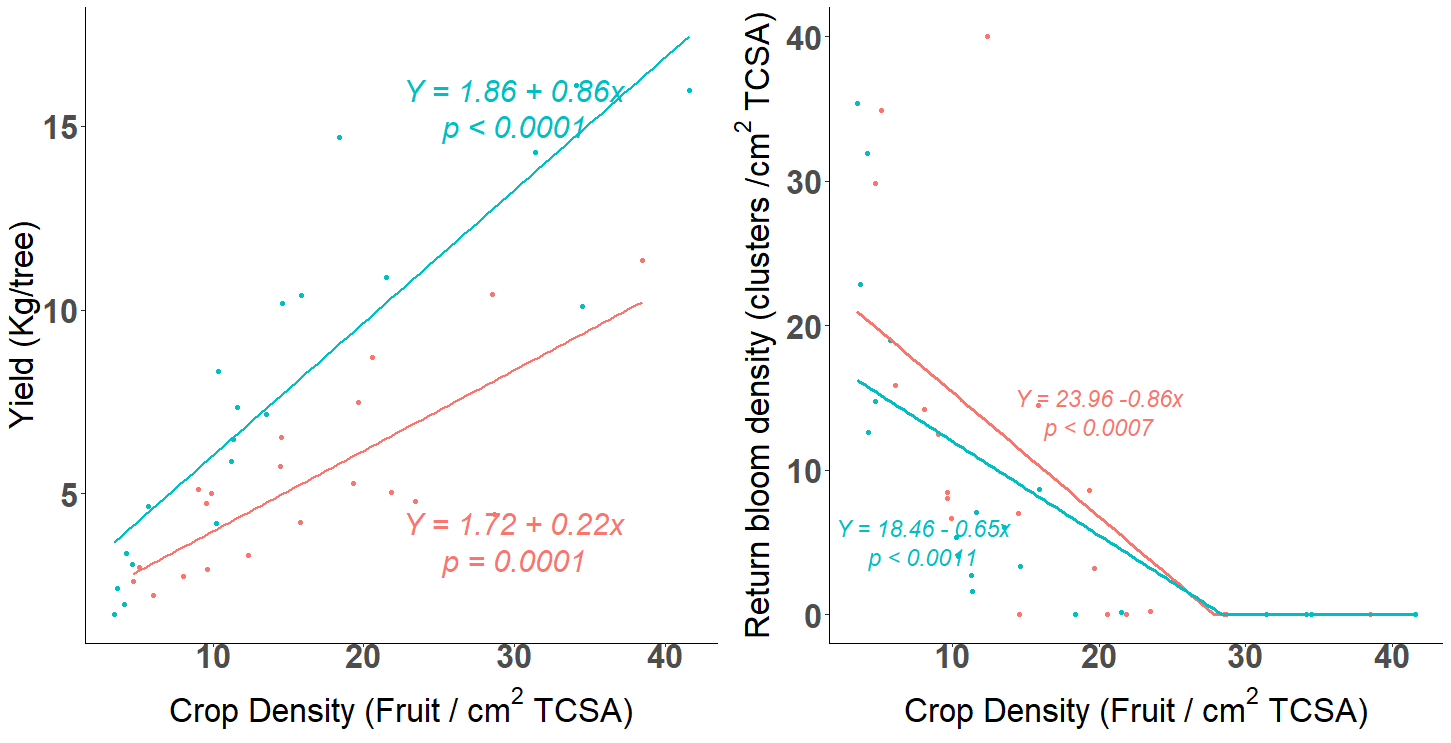


**Supplementary Figure 1.** Regression between crop density and yield (A) and return bloom density (the following spring) (B) for ‘Porters Perfection’/‘G.11’ and ‘Binet Rouge’/‘G.11’ subjected to four crop density treatments. Each point represents a single measurement of one tree.
